# ID 271 - Perfil de Segurança da Miltefosina no Tratamento da Leishmaniose Tegumentar: uma análise do monitoramento em Minas Gerais

**DOI:** 10.5327/2237-9622.2025.v34s1.271

**Published:** 2025-11-25

**Authors:** Sarah Nascimento Silva, Laís Raquel Ribeiro, Janaína de Pina Carvalho, Mell Ferreira Saliba, Gláucia Cota

**Keywords:** leishmaniose, efeitos colaterais e reações adversas relacionados a medicamentos, toxicidade, miltefosina

## Abstract

**Introdução:**

A incorporação de uma nova tecnologia no sistema de saúde requer um grande planejamento, envolvendo ações para a sua implementação e monitoramento dos resultados de efetividade e da segurança na população em uso. A miltefosina é o primeiro e o único tratamento disponível na forma oral para leishmaniose, e foi distribuída no Sistema Único de Saúde (SUS) a partir de 2021. O objetivo do estudo foi avaliar o perfil de segurança da miltefosina no tratamento da leishmaniose tegumentar durante a fase inicial de implementação em Minas Gerais.

**Método:**

Foram analisados os dados de todos os pacientes com diagnóstico confirmado de leishmaniose submetidos a tratamento com miltefosina na fase-piloto de implementação desse novo tratamento. Foram elegíveis para essa análise aqueles que receberam ao menos uma dose de miltefosina e que tinham formulário de monitoramento disponível, ainda que incompleto. As fontes de dados foram as Fichas de Notificação de Leishmaniose Tegumentar do Sistema de Informação de Agravos de Notificação (Sinan), para análise de dados demográficos e clínicos; formulário de solicitação de miltefosina e formulário de monitoramento. Qualquer sinal/sintoma e anormalidade laboratorial novo ou agravado que surgisse após o início da terapia com miltefosina foi considerado como evento adverso (EA). Os EAs foram classificados de acordo com o (MedDRA), a gravidade foi definida segundo critérios da Organização Mundial da Saúde (OMS), e a intensidade dos sintomas com base na tabela da (DAIDS). Desordens oculares foram classificadas como eventos de interesse especial (EIE) após um alerta de segurança da OMS em 2023.

**Resultados:**

Um conjunto de 157 pacientes tratados com miltefosina no estado de Minas Gerais, entre julho de 2021 e agosto de 2023, foi elegível para essa análise. Foram identificados 316 EAs em 121 pacientes (77,1% dos casos), o que representa uma média de dois eventos por paciente tratado com miltefosina. Desse total, 229 foram EAs clínicos, com predomínio de manifestações relacionadas ao sistema gastrointestinal (174 eventos, 76%), seguido por distúrbios musculoesqueléticos e do tecido conjuntivo, com destaque para o relato de artralgia, descrito para 22 (14%) pacientes. Entre os 147 pacientes com resultados do monitoramento laboratorial disponível, 68 (46,3%) tiveram ao menos um parâmetro alterado durante o seguimento. A alteração laboratorial mais comum foi a elevação da creatinina sérica, significativamente associada com hipertensão arterial, idade e forma clínica mucosa. Não houve registros de gravidez entre mulheres tratadas com miltefosina durante o período de implementação-piloto. A taxa de interrupção precoce do tratamento foi de 11,8%, associada à idade e à presença de alteração basal de creatinina sérica. Metade dos pacientes teve interrupção temporária ou uso irregular do tratamento, necessitando mais de 28 dias para completar o tratamento programado.

**Conclusão:**

Esse modelo de estratégia de farmacovigilância trouxe úteis e representa uma abordagem com potencial para ser aplicada a novas tecnologias no contexto de outros programas de controle de doenças negligenciadas. A presença de EIE com o uso da miltefosina destaca a necessidade de atenção dos serviços e profissionais. Esses são dados essenciais para retroalimentar o sistema de gestão de tecnologias em saúde e subsidiar novas decisões, contribuindo para a sustentabilidade do sistema de saúde e para a segurança do paciente.

